# Ripasudil in a Model of Pigmentary Glaucoma

**DOI:** 10.1167/tvst.9.10.27

**Published:** 2020-09-25

**Authors:** Chao Wang, Yalong Dang, Susannah Waxman, Ying Hong, Priyal Shah, Ralitsa T. Loewen, Xiaobo Xia, Nils A. Loewen

**Affiliations:** 1 University of Würzburg, Department of Ophthalmology, Würzburg, Germany; 2 University of Pittsburgh School of Medicine, Department of Ophthalmology, Pittsburgh, PA, USA; 3 Eye Center of Xiangya Hospital, Central South University, Changsha, Hunan, China; 4 Hunan Key Laboratory of Ophthalmology, Changsha, Hunan, China; 5 Sanmenxia Central Hospital, Sanmenxia, Henan, China

**Keywords:** pigment dispersion, anterior segment perfusion model, pigmentary glaucoma, rho-kinase inhibitor

## Abstract

**Purpose:**

To investigate the effects of Ripasudil (K-115), a Rho-kinase inhibitor, in a porcine model of pigmentary glaucoma.

**Methods:**

In vitro trabecular meshwork (TM) cells and ex vivo perfused eyes were subjected to pigment dispersion followed by K-115 treatment (PK115). PK115 was compared to controls (C) and pigment (P). Cytoskeletal alterations were assessed by F-actin labeling. TM cell phagocytosis of fluorescent targets was evaluated by flow cytometry. Cell migration was studied with a wound-healing assay. Intraocular pressure was continuously monitored and compared to after the establishment of the pigmentary glaucoma model and after treatment with K-115.

**Results:**

The percentage of cells with stress fibers increased in response to pigment but declined sharply after treatment with K-115 (P: 32.8% ± 2.9%; PK115: 11.6% ± 3.3%, *P* < 0.001). Phagocytosis first declined but recovered after K-115 (P: 25.7% ± 2.1%, PK115: 33.4% ± 0.8%, *P* <0.01). Migration recuperated at 12 hours with K-115 treatment (P: 19.1 ± 4.6 cells/high-power field, PK115: 42.5 ± 1.6 cells/high-power field, *P* < 0.001). Ex vivo, eyes became hypertensive from pigment dispersion but were normotensive after treatment with K-115 (P: 20.3 ± 1.2 mm Hg, PK115: 8.9 ± 1.7 mm Hg; *P* < 0.005).

**Conclusions:**

In vitro, K-115 reduced TM stress fibers, restored phagocytosis, and restored migration of TM cells. Ex vivo, K-115 normalized intraocular pressure.

**Translational Relevance:**

This ex vivo pigmentary glaucoma model provides a readily available basis to investigate new drugs such as the rho-kinase inhibitor studied here.

## Introduction

Pigment dispersion syndrome (PDS) is characterized by the shedding of pigmented debris from the iris pigment epithelium and its ectopic accumulation throughout the anterior segment of the eye but primarily in the trabecular meshwork (TM).^1^ Pigmentary glaucoma, a common secondary open-angle glaucoma, can develop as a result of PDS. It is characterized by heavy pigmentation of the TM, increased intraocular pressure (IOP), and characteristic glaucomatous optic neuropathy.^2^ An increased outflow resistance results from an altered TM cytoskeleton, a decline in phagocytosis and migration, and activation of the Rho signaling pathway as can be shown both in vitro^3^ and ex vivo.^4^

Beta-blockers and prostaglandins effectively lower IOP in pigmentary glaucoma.^5^^,^^6^ Miotics lower IOP and reduce the underlying pigment dispersion by minimizing friction between zonules and the iris. However, this is poorly tolerated in nonpresbyopic patients because of myopization.^7^ Rho-kinase inhibitors are a more recent class of glaucoma medications that increase the outflow facility by relaxing the TM cytoskeleton^8^^–^^11^ and dilating the distal outflow tract.^12^ The cytoskeleton plays an important role in TM-mediated regulation of AH outflow. Ripasudil (K-115) inhibits both isoforms of rho-associated, coiled-coil–containing protein kinase (ROCK), ROCK-1, and ROCK-2.^13^ K-115 is an isoquinoline monohydrochloride dihydrate that binds to the ATP-dependent kinase domain in the hydrophobic cleft between the N- and C-terminal lobes of the kinase domain,^14^ just like fasudil^15^ from which it is derived. It has an IC_50_ for ROCK 1 and ROCK 2 of 51 and 19 nmol/L, respectively. Tanihara et al.^16^ found that a 0.4% solution of K-115 lowered the IOP by around 20% from a low baseline of 19 mm Hg. In this study, we hypothesized that K-115 can reverse pathological changes of the TM induced by pigment dispersion.

## Materials and Methods

### Pig Eyes and Primary Trabecular Meshwork Cells

Porcine eyes were obtained from a local abattoir (Thoma Meat Market, Saxonburg, PA, USA) and processed within two hours of sacrifice. TM cells that were obtained and characterized by assays as described previously in greater detail and is summarized in the following.^3^ TM cells used in this study came from the same pool of eyes. Briefly, after decontamination and dissection, TM tissues were carefully excised by ab interno trabeculectomy under a surgical microscope, isolated in fragments, and allowed to grow out. Only the first four passages of primary TM cells were used for this study. TM cells were immunostained with preferential TM-marker antibodies to validate their identity including anti-MGP, anti-alpha-SMA, anti-AQP1. Induction of myocilin by dexamethasone confirmed their typical TM cell behavior and measured by quantitative polymerase chain reaction.

### Generation of Pigment

The generation of pigment followed our previous protocol.^3^ Irises were collected and frozen at −80°C for two hours and thawed at room temperature. Freeze-thaw was repeated twice. Irises were added to 15 ml of phosphate-buffered saline solution (PBS), agitated to produce pigment particles, and filtered through a 70-µm cell strainer (431751, Corning Incorporated, Durham, NC, USA). Particles were washed three times and suspended in 4 ml of PBS as a stock solution.

### Pigmentary Glaucoma Model and K-115 Treatment

The pigmentary glaucoma model was established as previously described.^3^ Briefly, 24 paired pig eyes were obtained from a local meat market within two hours of sacrifice. The extraocular tissues were removed carefully. After disinfecting, the iris, lens, ciliary body, and posterior segment were removed. Anterior segments were mounted in perfusion dishes and perfused with Dulbecco's modified Eagle's medium supplemented with 1% fetal bovine serum (FBS, 10438026; Thermo Fisher Scientific, Waltham, MA, USA) and 1% antibiotics (15240062, Thermo Fisher Scientific) at 3 µL/min. IOPs were recorded every two minutes with transducers (SP844; MEMSCAP, Skoppum, Norway) using LabChart software (AD Instruments, Colorado Springs, CO, USA). After 48 hours, the media was supplemented with either 1.67  ×  10^7^ particles/mL of pigment granules (P, n = 8), pigment plus 10 µmol/L K-115 (PK115, n = 7), or vehicle control consisting of normal perfusion medium (C, n = 8). The number of pigment granules had been established before^4^ and used several times^3^^,^^10^^,^^17^^,^^18^ to induce ocular hypertension with this protocol.

### Actin Cytoskeleton Staining of Primary TM Cells

For all in vitro experiments, TM cells were seeded into six-well plates at a concentration of 1 × 10^5^ cells/well. Pigment media contained 1.67  ×  10^7^ particles/mL. Stress fiber formation was evaluated through the labeling of F-actin. TM cells were seeded into one six-well plate with a coverslip placed at the bottom of each well. After reaching 80% confluency, six wells were treated with pigment media, and three wells were treated with vehicle control. After 24 hours, three of the pigment treated wells were additionally treated with 10 µmol/L K-115 for 24 hours. Cells were fixed with 4% PFA for one hour at room temperature and washed with PBS three times. Samples were incubated with Alexa Fluor 488 Phalloidin (1:60 dilution, A12379, Thermo Fisher, Waltham, MA) for 30 minutes. DAPI was used to stain cell nuclei. Ten images of each group were acquired with an upright laser scanning confocal microscope (info) at magnification ×600. The percentage of cells with stress fibers in each image was calculated.

### TM Phagocytosis Assay

TM phagocytosis was determined by flow cytometry. Three wells were treated with control media and six wells were treated with pigment media. After 24 hours, three of the pigment treated wells were additionally treated with 10 µmol/L K-115 for 24 hours. After washing with PBS three times, cells were incubated with 0.5 µm fluorescein isothiocyanate (FITC)-labeled microspheres (F8813, Thermo Fisher, Waltham, MA) at a concentration of 5 × 10^8^ microspheres/mL for one hour at 37°C. Wells were rinsed with PBS three times, cells digested with trypsin, resuspended in 200 µL PBS, and filtered with a 70-µm cell strainer. Flow cytometry was performed to evaluate the percentage of cells that had phagocytosed FITC-labeled microspheres.

### Wound-Healing Assay

The ability of TM cells to migrate was assessed with a wound-healing assay. Nine wells were seeded, allowed to reach 100% confluency, and then treated with 10 µg/mL mitomycin (M4287; Sigma Aldrich, ST. Louis, MO, USA) for one hour to inhibit cell division. Wells were washed with PBS three times, and a cell-free gap was created with a single pass of a 10-µL pipette tip (F1732031; Gilson, Middleton, WI, USA). Floating cells were gently washed away with prewarmed media. Three wells were treated with pigment media, three wells were treated with pigment media supplemented with 10 µmol/L K-115, and three wells were treated with vehicle control. Cells were cultured in a microscope stage incubator (H301-TC1-HMTC; Okolab, S.r.L., Ottaviano, Italy), and images were taken at magnification ×40, every six hours for 48 hours using a live cell microscope system (Nikon Eclipse TI-E; Nikon, Tokyo, Japan). Cells that migrated into the cell-free gap were counted in 10 high-power fields.

### Histology

Anterior segments were dismounted from the perfusion dishes and washed with PBS. Tissues were fixed with 4% PFA for 24 hours and embedded in paraffin. Samples were sectioned at 6-µm thickness, followed by staining with hematoxylin and eosin (H&E).

### Statistics

All quantitative data were described as the mean ± standard error (SE) and analyzed using PASW Statistics 18.0 (SPSS Inc., Chicago, IL, USA). A *P* value ≤ 0.05 was considered statistically significant. We used a paired *t*-test to compare same-eye IOP changes from baseline and one-way analysis of variance to compare several groups. Effect sizes were calculated using Cohen's d.

## Results

### IOP Reduction

**Figure 1.**
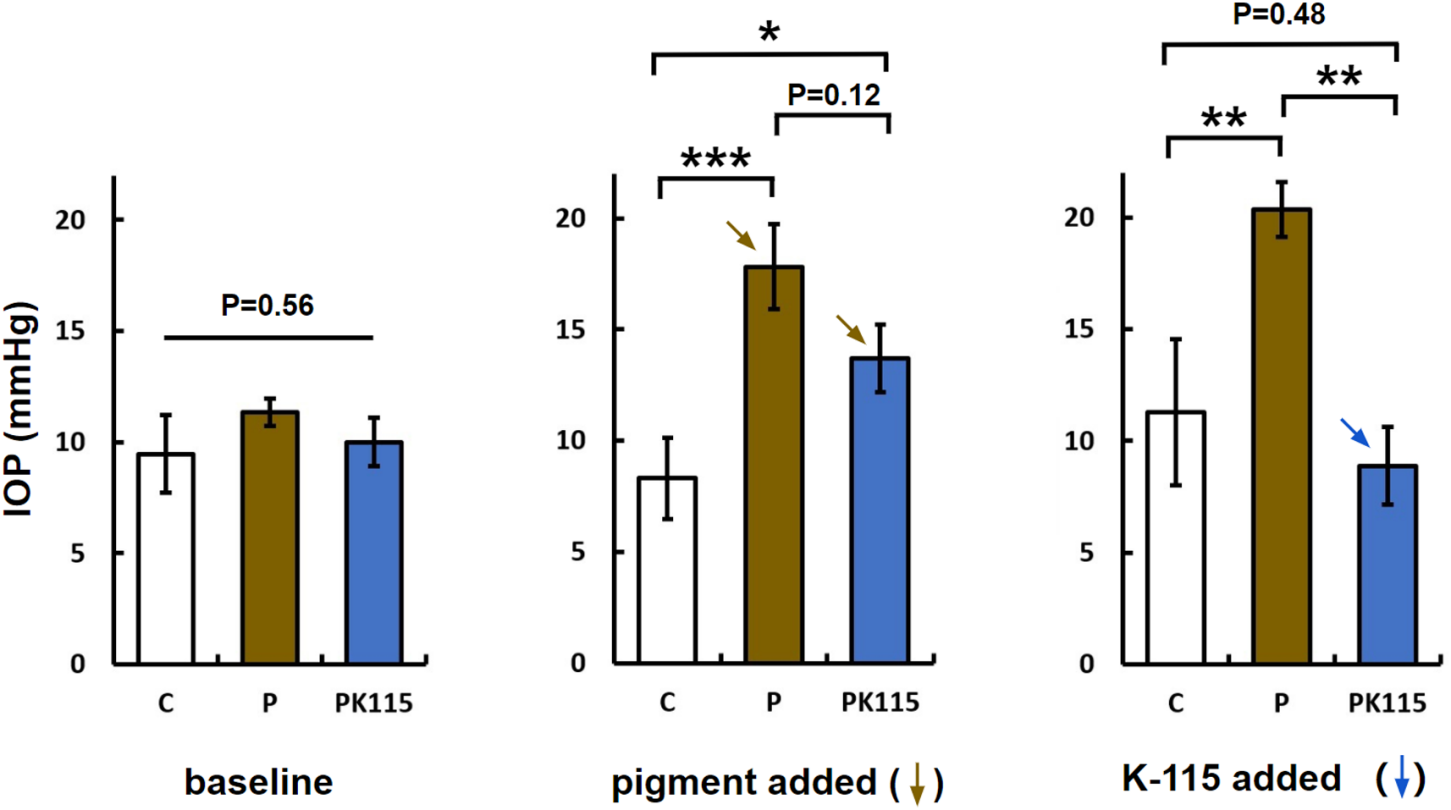
Baseline IOP of C (*white*), P (*brown*), and PK115 (*blue*) were similar (*P* = 0.56), rose in P (***P < 0.001) and PK115 (*P < 0.01) after pigment was added to the perfusion. After starting K-115 in PK115, IOP dropped to the level of control (difference C and PK115: *P* = 0.48).

Baseline IOPs of the three groups of eyes were very similar after48 hours of stabilization (C: 9.5 ± 1.8 mm Hg, n = 8; P: 11.3 ± 0.6 mm Hg, n = 8; PK115: 10.0 ± 1.1 mm Hg, n = 7, P = 0.56, Fig. 1). After pigment treatment for 48 hours, IOP increased in P by 114% to 17.8 ± 1.6 mm Hg (compared to C: 8.3 ± 1.8 mm Hg, *P* < 0.001). Before adding K-115, IOP increased in PK115 by 65% to 13.7 ± 1.5 mm Hg (compared to C, *P* < 0.01). There was no difference between P and PK115 (*P* = 0.12). IOP of C remained unchanged (*P* = 0.65). After 48 hours of K-115, IOP in PK115 had dropped compared to P (PK115: 8.9 ± 1.7 mm Hg, P: 20.3 ± 1.2 mm Hg, *P* < 0.005). IOP of C and PK-115 were similar (11.2 ± 3.3 mm Hg, n = 8, *P* = 0.48). One eye in the PK115 group was excluded because of a contamination.

### TM Morphology

**Figure 2.**
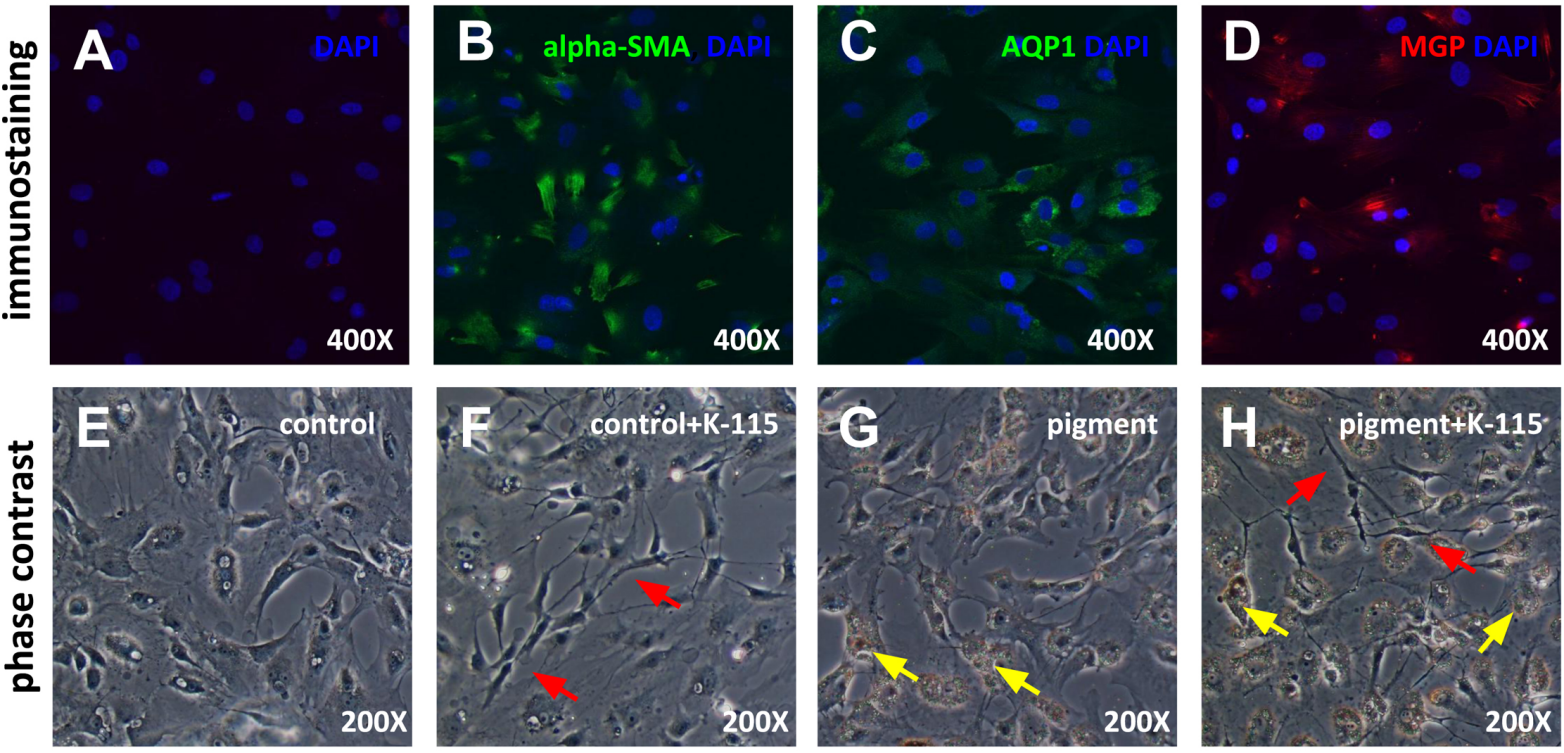
Primary TM cells nuclei of control group were labeled with DAPI (A). Primary TM cells expressed the TM markers alpha-SMA (B), AQP1 (C), and MGP (D). Compared to control (E), cells treated with K-115 at 10 µmol/L contracted (F, H, *red arrow*). Pigment granules were phagocytosed by TM cells (G, H, *yellow arrows*).

Three markers expressed in TM cells were selected for immunohistochemical analysis of TM primary culture: alpha-SMA, AQP1, and MGP. TM cells were positive for all three TM markers. Primary TM cells were treated with pigment media for 24 hours and observed under a phase-contrast microscope. TM cells formed a monolayer of flat and polygonal, cobblestone-like cells as expected. Pigment particles visible in P were phagocytosed and accumulated in the cells (Fig. 2, yellow arrows). After treatment with K-115, TM cell bodies contracted and formed long, thin processes (Fig. 2, red arrows).

### Disruption of Actin Stress Fibers

**Figure 3.**
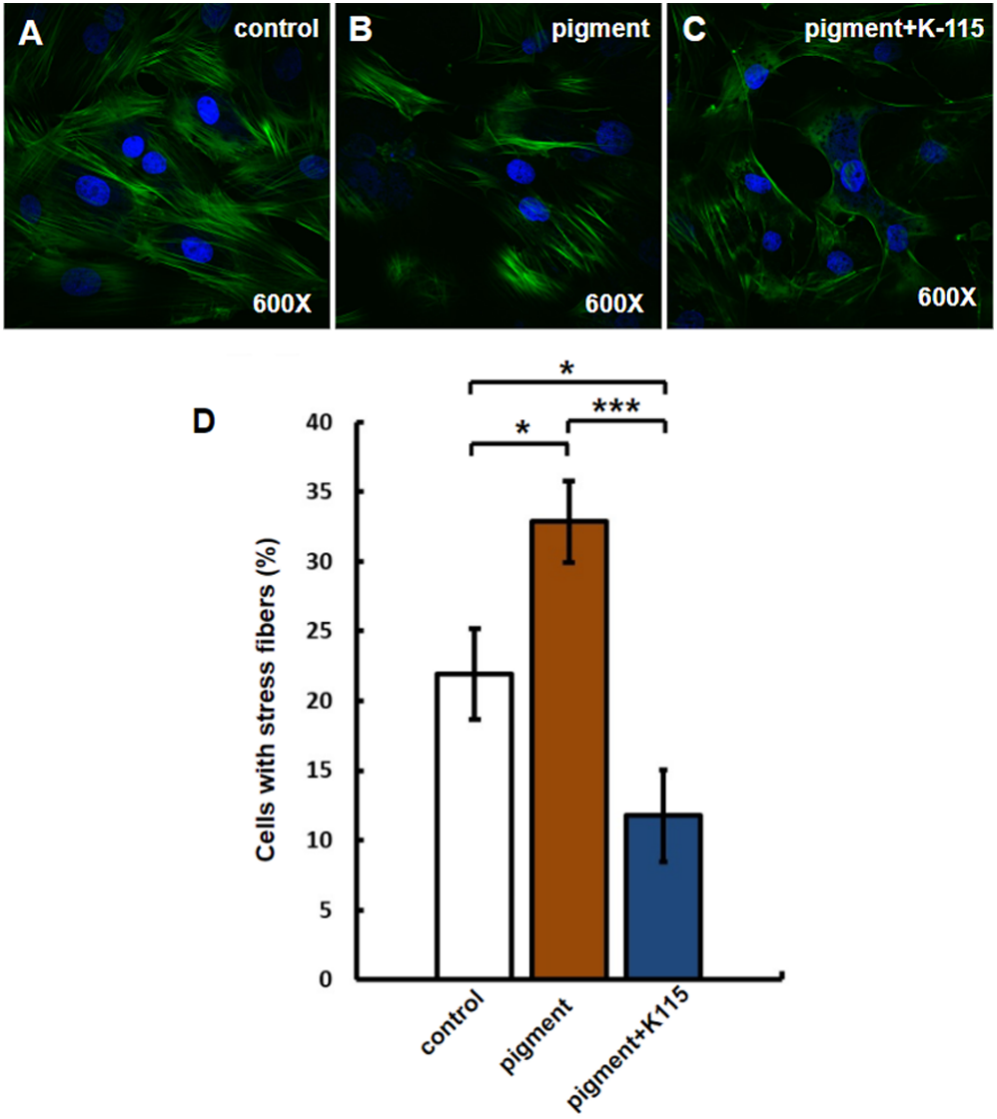
Compared with C (21.9% ± 3.2%, n = 10 visual fields of three wells, * *P* < 0.05), P (32.8% ± 2.9%, n = 10 visual fields of three wells, * *P* < 0.05) had a higher proportion of cells containing stress fibers. After K-115 treatment, TM cells containing stress fibers were reduced (C, *white arrows*, P: 32.8% ± 2.9%; PK115: 11.6% ± 3.3%, n = 10 visual fields of three wells, *** *P* < 0.001).

F-actin was labeled with Alexa Fluor 488 conjugated phalloidin. In C, F-actin was thin and smooth (Fig. 3). After 48 hours of pigment treatment, F-actin in P showed thick, long, and curved stress fibers. The percentage of cells that contained stress fibers in P (32.8 ± 2.9%, n = 10 visual fields of three wells) was higher than that in C (21.9% ± 3.2%, n = 10 visual fields of three wells, *P* < 0.05). After 24 hours of pigment treatment, PK115 was treated with 10 µmol/L K-115 for another 24 hours. After treatment with K-115, the percentage of cells with stress fibers was reduced (PK115: 11.6 ± 3.3%, n = 10 visual fields of three wells, *P* < 0.001).

### Increased Phagocytosis

**Figure 4.**
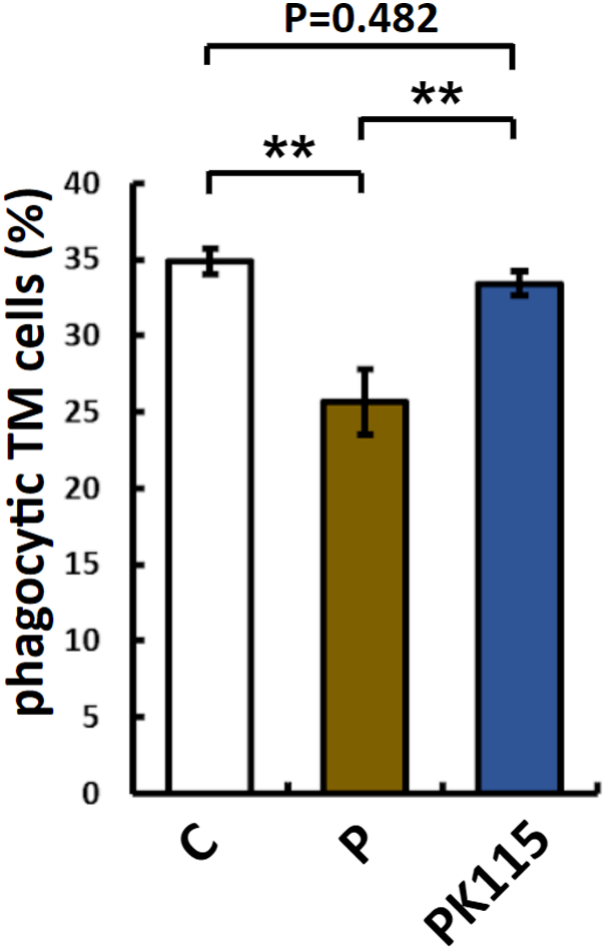
After 48 hours of pigment treatment, the proportion of TM cells that contained fluorescent microspheres in *P* was lower than that in *C* (C: 34.9% ± 0.8%, P: 25.7% ± 2.1%, n = 3 wells, ** *P* < 0.01). After 24 hours of K-115 treatment in pigment-treated cells, the proportion of cells that phagocytosed fluorescent microspheres was higher than that in *P* (P: 25.7.0% ± 2.1%, PK115: 33.4% ± 0.8%, n = 3 wells, ** *df* < 0.01).

After 48 hours of pigment treatment, the proportion of TM cells that phagocytosed fluorescent microspheres in P was lower than C (C: 34.9% ± 0.8%, P: 25.7% ± 2.1%, n = 3 wells, *P* < 0.01, Fig. 4). After 24 hours of pigment treatment, PK115 was treated with 10 µmol/L K-115 for 24 hours. The proportion of PK115 TM cells that phagocytosed fluorescent microspheres was higher than P and not different from C (P: 25.7.0 ± 2.1%, PK115: 33.4 ± 0.8%, n = 3 wells, *P* < 0.01).

### Increased Cell Motility

**Figure 5.**
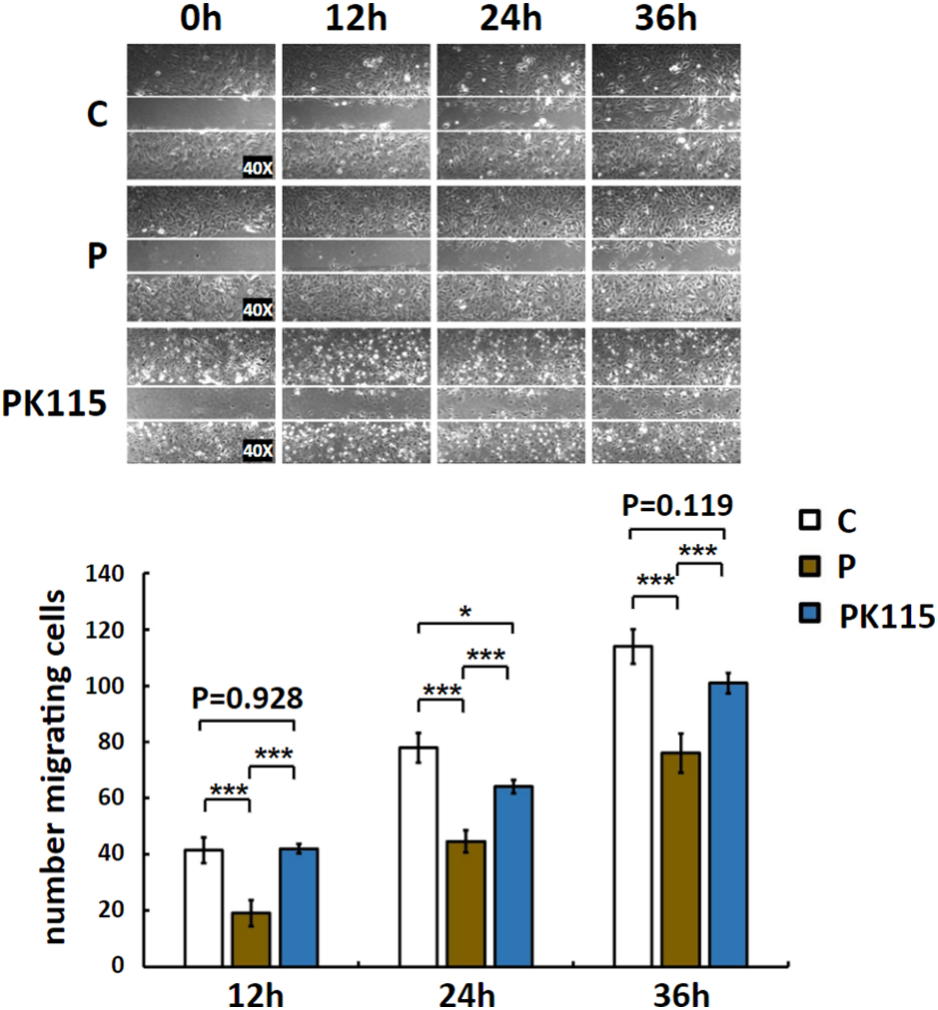
The number of TM cells that migrated to the cell-free wound in P at 12 hours (C: 41.5 ± 4.6, P: 19.1 ± 4.6, n = 10 visual fields of three wells, *** *P* < 0.001), 24 hours (C: 77.9 ± 5.2), P: 44.6 ± 4.0, n = 10 visual fields of three wells, *** *P* < 0.001), and 36 hours (C: 114.0 ± 6.1, P: 76.0 ± 6.9, n = 10 visual fields of three wells, *** *P* < 0.001) was less than C; and K-115 increased the number of TM cells migrating to the cell-free zone at 12 hours (P: 19.1 ± 4.6, PK115: 42.5 ± 1.6, n = 10 visual fields of three wells, *** *P* < 0.001); 24 hours (P: 44.6 ± 4.0, PK115: 64.1 ± 2.3, n = 10 visual fields of three wells, *** *P* < 0.001); and 36 hours (P: 76.0 ± 6.9, PK115: 100.9 ± 3.7, n = 10 visual fields of three wells, *** *P* < 0.001).

A wound-healing experiment was used to assess the migratory capacity of TM cells. The number of TM cells that migrated into the cell-free wound was counted at 12, 24, and 36 hours after pigment treatment. TM cells gradually migrated into the cell-free zone at 12, 24, and 36 hours after pigment treatment. TM cells had a lower migration in P than in C at all time points (12 hours: C: 41.5 ± 4.6; P: 19.1 ± 4.6, *P* < 0.001; 24 hours: C: 77.9 ± 5.2, P: 44.6 ± 4.0, *P* < 0.001; 36 hours: C: 114.0 ± 6.1, P: 76.0 ± 6.9, n = 10 visual fields of three wells, *P* < 0.001, Fig. 5). Compared with P, PK115 had higher levels of migration into the wound site at each time point as well (12 hours: P: 19.1 ± 4.6, PK115: 42.5 ± 1.6, *P* < 0.001 24 hours: P: 44.6 ± 4.0, PK115: 64.1 ± 2.3, *P* < 0.001; 36 hours: P: 76.0 ± 6.9, PK115: 100.9 ± 3.7, n = 10 visual fields of three wells, *P* < 0.001).

### Histology

**Figure 6.**
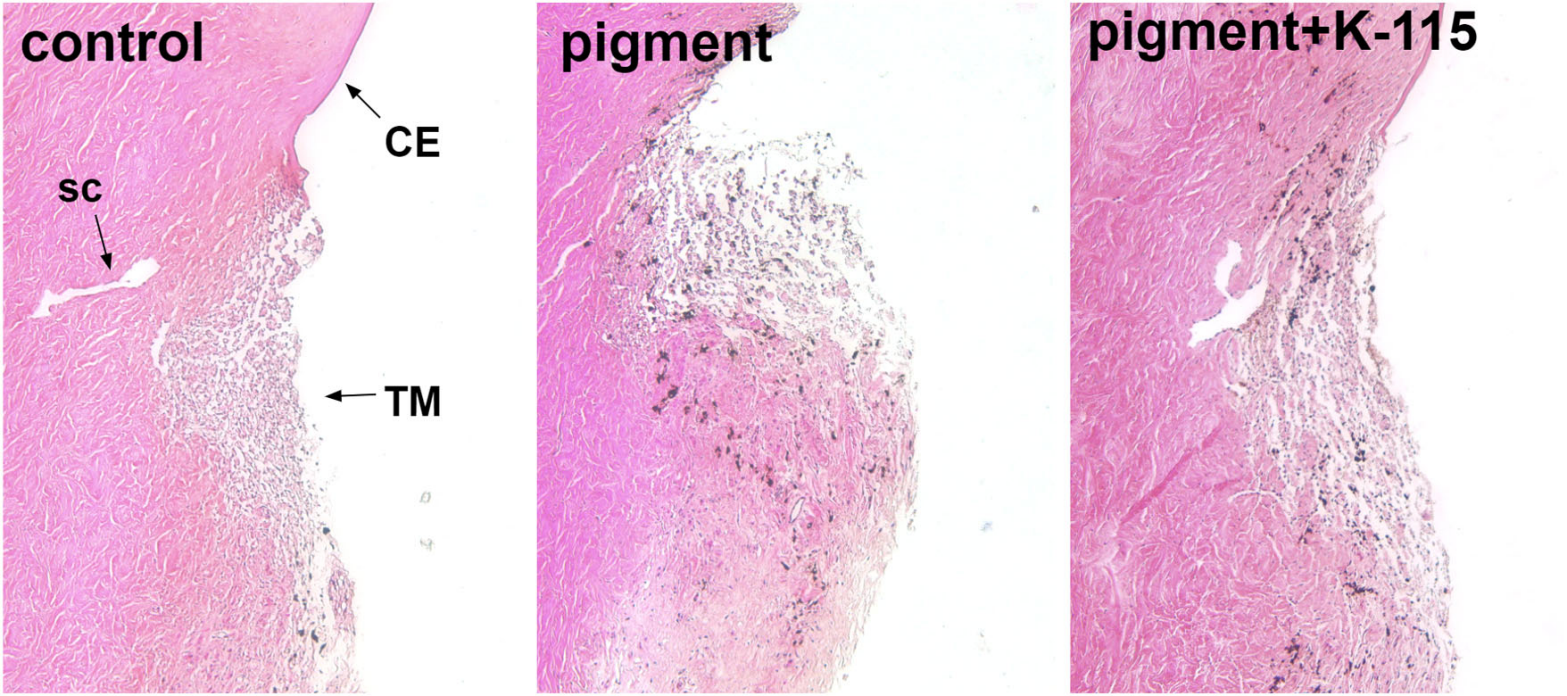
Examples of TM histology of controls (*left*), pigment (*middle*), and pigment+K-115 (*right*) group presented as a lightly pigmented strainer-like tissue. TM of pigment dispersion eyes showed phagocytosed pigment more common (*red arrows*). No histologic change was observed after K-115 treatment. SC, Schlemm's canal; CE, corneal endothelium.

The control TM appeared as a thick, multilayered structure that was lightly pigmented (Fig. 6). After perfusion with pigment-supplemented media for 48 hours, the pigment could be seen deep in the angular aqueous plexus. The effect size of K-115 on IOP was 3, on stress fibers 7, on phagocytosis 5, and on motility 5, all of which exceeded Cohen's descriptors for effect magnitude as “large”^19^ but matched Sawilowsky as “huge”.^20^

## Discussion

Pigmentary glaucoma is caused by the dispersion of pigment from the posterior side of the iris and ciliary processes that accumulates in the TM. Dysfunction of TM leads to increased outflow resistance and elevated IOP. In earlier studies with primary porcine TM cells^3^ and porcine anterior segment perfusion model,^4^ we found that pigmentary debris caused an increase of TM cell stress fibers, reduced phagocytosis, and mobility, and led to a persistent IOP elevation.^17^ Through gene microarray and signal pathway analysis, we found that the Rho pathway was activated in TM cells.^3^^,^^4^ Here, we investigated whether the Rho kinase inhibitor K-115 would reverse some of these changes both in vitro and ex vivo.

Several studies found that Rho-kinase inhibitors Y-27632, Y-39983, HA-1077, and H-1152, could increase outflow and decrease IOP by altering the TM cytoskeleton.^8^^,^^21^^–^^24^ Stress fibers can contract^25^ and increase stiffness^26^^,^^27^ leading to decreased outflow in primary open-angle glaucoma^28^ and also in glucocorticoid-induced glaucoma.^29^ In our study, pigment dispersion caused a marked increase in stress fibers and IOP but then dropped to baseline after K-115 treatment.

The TM removes debris by phagocytosis^30^ and has the ability to present antigens to the immune system.^31^ It has been observed that porcine TM cells phagocytose pigment granules within a few hours. When the pigment concentration exceeds a certain threshold, TM phagocytosis will decrease.^3^^,^^4^ Similarly, this study showed that phagocytosis of TM cells decreases after pigment treatment and but that K-115 promotes fluorescent microsphere intake again.

Several studies found that reduced TM cell mobility may cause IOP elevation. Fujimoto et al. reported that dexamethasone-induced cross-linked actin networks (CLANs) and decreased cell migration.^32^ Y-27632 counteracted the formation of CLANs. Clark found that Y-27632 lowered IOP and promoted TM cell migration in rabbits^9^ similar to our observation that K-115 recovered TM mobility and IOP. It increased endothelial cell migration^33^ above normal, but in our study, did not change TM cell migration above baseline.

We have previously examined RKI-1447 in the model used here. Although RKI-1447 is a pyridyl thiazolyl-urea structurally different from K-115, an isoquinoline, it is also a type I kinase inhibitor that binds the ATP binding site through interactions with the hinge region and the DFG motif and inhibits both ROCK1 and ROCK2.^34^ It has a half-maximal inhibitory concentration (IC_50_) of 14.5 nmol/L and 6.2 nmol/L for ROCK1 and ROCK2, respectively. In our studies, RKI-1447 achieved an IOP reduction of 33.6%. Although the present study suggests a larger IOP reduction of 50% for K-115, a side by side comparison to RKI-1447 would be helpful. RKI-1447 and K-115 have a similar specificity for ROCK 1 over ROCK2, but RK-1447 has no effect on the phosphorylation levels of AKT, MEK, and S6 kinase even at high concentrations.

In conclusion, K-115, a ROCK inhibitor, decreased TM stress fibers, increased phagocytosis, and motility in vitro, and lowered IOP ex vivo in a model of PG.
